# Tradeoffs in the value of biodiversity feature and cost data in conservation prioritization

**DOI:** 10.1038/s41598-019-52241-2

**Published:** 2019-11-04

**Authors:** Amanda D. Rodewald, Matt Strimas-Mackey, Richard Schuster, Peter Arcese

**Affiliations:** 1 Cornell Lab of Ornithology, 159 Sapsucker Woods Rd., Ithaca, NY 14850 USA; 2 Department of Natural Resources, Cornell University, Ithaca, NY 14853 USA; 3 Department of Biology, 1125 Colonel By Drive, Carleton University, Ottawa, ON K1S 5B6 Canada; 4 Ecosystem Science and Management Program, 3333 University Way, University of Northern British Columbia, Prince George, BC V2N 4Z9 Canada; 5 Department of Forest and Conservation Sciences, University of British Columbia, Vancouver, BC V6T 1Z4 Canada

**Keywords:** Conservation biology, Ecological modelling, Ecology, Biodiversity, Conservation biology

## Abstract

Decision-support tools are commonly used to maximize return on investments (ROI) in conservation. We evaluated how the relative value of information on biodiversity features and land cost varied with data structure and variability, attributes of focal species and conservation targets, and habitat suitability thresholds for contrasting bird communities in the Pacific Northwest of North America. Specifically, we used spatial distribution maps for 20 bird species, land values, and an integer linear programming model to prioritize land units (1 km^2^) that met conservation targets at the lowest estimated cost (hereafter ‘efficiency’). Across scenarios, the relative value of biodiversity data increased with conservation targets, as higher thresholds for suitable habitat were applied, and when focal species occurred disproportionately on land of high assessed value. Incorporating land cost generally improved planning efficiency, but at diminishing rates as spatial variance in biodiversity features relative to land cost increased. Our results offer a precise, empirical demonstration of how spatially-optimized planning solutions are influenced by spatial variation in underlying feature layers. We also provide guidance to planners seeking to maximize efficiency in data acquisition and resolve potential trade-offs when setting targets and thresholds in financially-constrained, spatial planning efforts aimed at maximizing ROI in biodiversity conservation.

## Introduction

Conservation decision-makers often respond to constraints on human effort, funding, or data quality by seeking to acquire land with high biodiversity values but low transaction, management, or opportunity costs. Although opportunism can play a role in such efforts^1^, decisions that fail to incorporate reliable ecological or cost data can increase the risk of protecting land with limited conservation value or high associated costs^2–5^. These shortcomings reduce the efficiency of conservation actions. Consequently, many decision-support tools now incorporate ecological design concepts, such as complementarity and irreplaceability, to identify portfolios of sites that efficiently conserve mapped biodiversity features while also minimizing the anticipated costs of land acquisition and/or management^6–10^.

Most recently, systematic spatial planning tools have employed integer linear-programming (ILP) to allow planners to include multiple features across a wide range of temporal and spatial scales to identify cost-effective solutions to highly complex conservation planning problems (e.g.^11–13^). However, whereas 96% of spatial prioritizations reviewed by Sinclair *et al*.^14^ included data on focal species, far fewer included spatial data on land value (24%) or implementation costs (33%). Such exclusions could arise due to the high cost of data acquisition, uncertainty about its precision, or a reluctance to make existing models more complex. Irrespective of the cause, these exclusions raise questions about the opportunity costs of improving biodiversity feature data versus spatial data on land or management costs and highlight inherent trade-offs in data type and quality that can affect long-term rates of return on funds and effort investment in conservation^2,5^. Theoretical studies suggest that the marginal value of biodiversity and land cost data in spatially-optimized conservation plans can vary with data structure and variability, the attributes of focal species, and the conservation targets or habitat suitability thresholds applied, with potentially dramatic effects on return on investment^5,15^. However, few empirical studies have examined this issue explicitly^1,2,6,16^.

Theory suggests that the risk of prioritizing low-value sites increases as spatial variation in costs exceed spatial variation in the ecological or other features of interest, and empirical studies suggest this situation is common and sometimes extreme (but see^17–22^). A corollary of this theory is that as the spatial variation of one feature layer becomes large relative to others, the more variable layer increasingly drives solutions^19,23^. However, despite the potential influence of spatial variation in biodiversity feature or cost data on the solutions obtained, empirical tests of these theoretical predictions are scarce (but see 5 for case study in the Appalachians and 15 for simulation study). In particular, few studies quantify the contribution of biodiversity feature data on the efficiency of optimized solutions or identify conditions under which ‘informed opportunism’ in area-based conservation plans is most likely to be achieved^24^.

In this paper, we estimate the value of biodiversity feature and land cost data on the efficiency of systematic conservation plans to protect focal birds of the Pacific Northwest of North America. Specifically, we examined how the relative value of cost and biodiversity data varied with (1) data structure and variability, (2) species attributes, (3) conservation targets, (4) and decision rules regarding acceptable levels of habitat suitability (Table 1). Because our study aimed to elucidate general principles underlying efficient conservation planning, rather than to identify a portfolio for real-world implementation, we focused our examination and findings using two groups of birds indicative of land of relatively low versus high cost, and associated with forested versus human-dominated landscapes, respectively.

**Table 1:** Range of values or conditions evaluated across prioritization scenarios.

Attribute	Values
Relative variability of data	CV of biodiversity data were 2, 4, 8, or 16 times that of cost data
Conservation target	0–100% of populations protected
Habitat suitability (occupancy threshold)	A species has a 25%, 50%, or 75% probability of occurrence
Species group	Forest vs. human-associated birds
Land cost	Incorporated vs not incorporated
Biodiversity data	Included vs. not included

## Methods

### Study area

**Figure 1:**
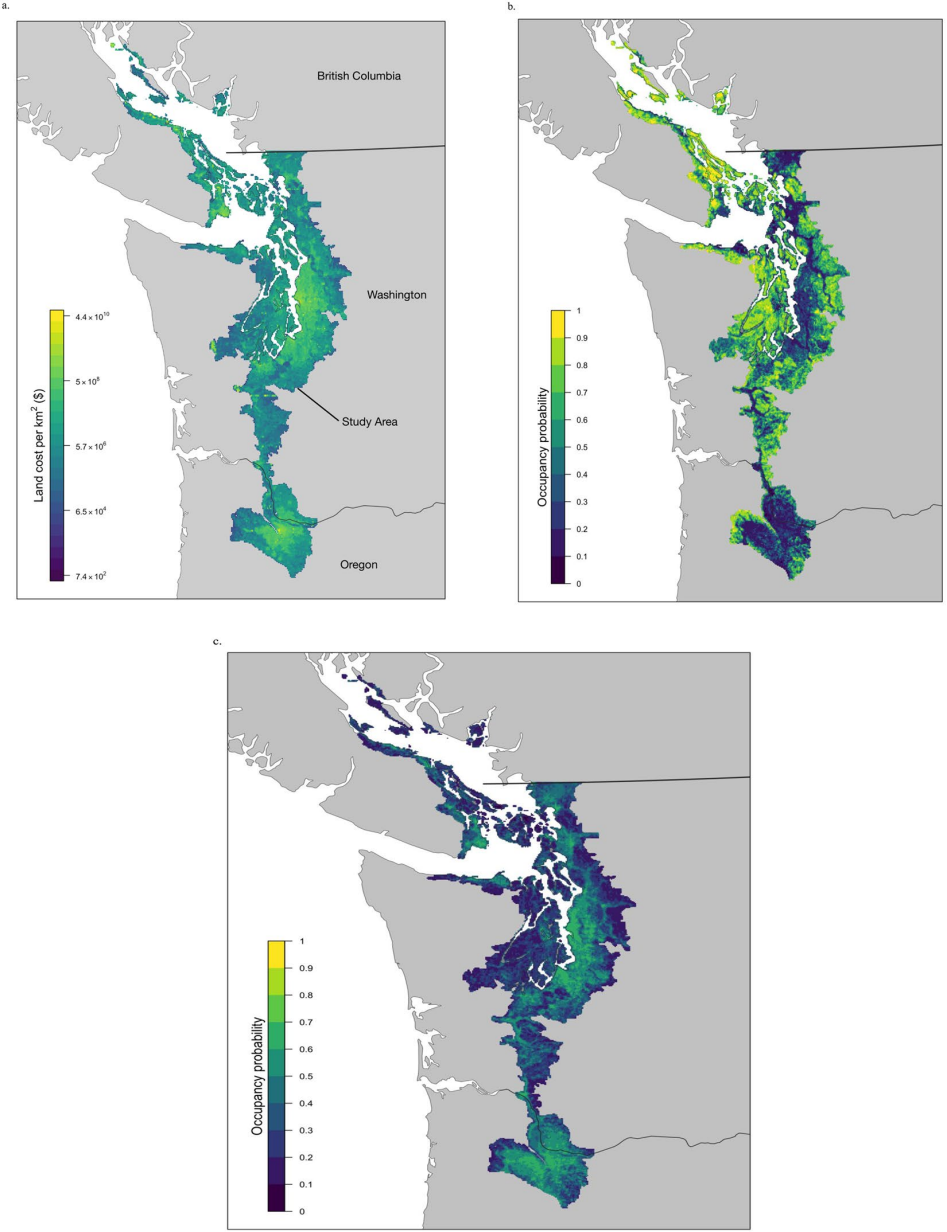
(**a**–**c**) The focal area for this study showing the (**a**) cost and occupancy probabilities for (**b**) forest-associated birds and (**c**) human-associated birds within 1 km^2^ planning units. Maps were produced using R version 3.6.1 (https://www.r-project.org/) with the following packages: fields v9.8-6, raster v3.0-2, rnaturalearth v0.1.0, sf v0.7-7, viridis v0.5.1. The base map is within the Creative Common’s domain and accessible through Natural Earth (https://www.naturalearthdata.com/).

We focused on a 27,250 km^2^ portion of the Georgia Basin, Puget Trough and Willamette Valley of Pacific Northwest of the US and Canada (Fig. 1), and experiencing climatic conditions typical of Coastal Douglas-fir (CDF) forest and savanna habitats of southwestern British Columbia^24^. Land cover in the region is diverse, with approximately 57% of the land in forest, 8% in savanna or grassland, 5% in cropland, and 10% being urban or built.

### Data layers

#### Biodiversity data

Our prioritizations were run with data from the eBird program, which is a citizen-science effort that has produced one of the largest and rapidly growing biodiversity databases in the world^25,26^. From the 2013 eBird Reference Dataset (http://ebird.org/ebird/data/download) we used a total of 12081 checklists in our study area, then filtered these checklists to retain only those from March – June to capture the breeding season, <1.5 hours in duration, <5 km travelled, and a maximum of 10 visits to a given location (unpublished R code; Hochachka, pers. com.). Sampling locations <100 m apart were collapsed to one location, yielding 5470 checklists from 2160 locations, visited from 1–10 times and 2.53 times on average (Supplementary Materials Fig. 1). Following Schuster *et al*.^27,28^ we used a combination of quantitative models and expert elicitation to identify which species were associated either with forest habitat or with human-dominated habitat, such as built or residential land (Supplemental Material Methods, Supplementary Material Tables 1 and 2). Data and code used to generate occupancy maps can be found at a GitHub repository (https://github.com/ricschuster/Tradeoffs-biodiversity-cost).

#### Cadastral layer and land cost

We incorporated spatial heterogeneity in land cost^27,28^ in our plan by using cadastral data and 2012 land value assessments from the Integrated Cadastral Information Society of BC, resulting in 193,623 polygons for BC^27,28^. Cadastral data, including tax assessment land values from Washington State came from the University of Washington’s Washington State Parcel Database (https://depts.washington.edu/wagis/projects/parcels/; Version: StatewideParcels_v2012n_e9.2_r1.3; Date accessed: 2015/04/30), as well as San Juan County Parcel Data with separate signed user agreement. The combined cadastral layer included 1.92 M polygons. Cadastral data, including tax assessment land values from Oregon State had to be sourced from individual counties, which included Benton, Clackamas, Columbia, Douglas, Lane, Linn, Marion, Multnomah, Polk, Washington and Yamhill. The combined cadastral layer for Oregon included 605,425 polygons.

### Conservation prioritization

To assess the importance of biodiversity data, we compared prioritizations using both cost and biodiversity data to prioritizations using only cost. In both cases, the goal was to identify a set of planning units that captured a given percentage of each species’ total occupancy across the entire study region. When prioritizing sites with biodiversity data, we modeled the ‘minimum set problem’ in conservation planning wherein the goal is to minimize the cost of the solution whilst ensuring that all conservation targets are met. This objective is similar to that used in Marxan^9^. As such, we used a Marxan-like approach to find the minimum set of planning units that met the given occupancy targets ranging from 5–100% (in 5% increments) for the lowest possible cost. When prioritizing sites without biodiversity data, we used a C-rank approach (Supplementary Material Appendix A), whereby sites were selected from the cheapest to most expensive until occupancy targets were satisfied for all species. To explore the influence of constraints on habitat quality, we obtained optimal solutions to our spatial planning problem using three progressively more conservative thresholds for identifying suitable habitat (e.g., occupancy probability [p(occ)] ≥25%, ≥50%, or ≥75% likely to occupy a site). Doing so was achieved simply by excluding sites with estimated occupancy probability less than the threshold indicated. For all scenarios, we used 1 km^2^ planning units, generated by aggregating the species and cost data to this coarser resolution from the original 1-ha cells. Aggregation was accomplished by taking the sum of cost data and the mean of species data for all 1-ha cells within the larger 1 km^2^ cells.

The relative value of cost data was assessed by comparing prioritizations generated with both cost and biodiversity feature data, to prioritizations based only on the latter. The value of biodiversity feature data was estimated similarly, by comparing the cost of scenarios that included biodiversity data to those based only on cost (i.e., C-rank). In both cases, we solved the Marxan-like prioritization problem for occupancy targets ranging from 5–100%, in 5% increments, while maintaining a occupancy threshold ≥75% to ensure that only high quality habitat was selected. When using cost data we selected the cheapest set of planning units that met the occupancy targets; without cost data, we selected the smallest number of 1 km^2^ planning units that met habitat area and quality targets.

The above prioritizations were repeated for the 10 forest and 10 human-associated species to explore the consquences of spatial variation in cost, under the expectation that the more variable layer would be disproportionately influential on the prioritized solution. All prioritizations were run using the the prioritizr package^29^ in R^30^, which solves conservation prioritization problems exactly using integer linear programming. We solved all problems without a boundary length modifier term (BLM) and to within 1% of the optimal solution.

### Relative variation in costs and benefits

We explored how the relative variation in biodiversity and cost data drove prioritization solutions by examining scenarios in which the coefficient of variation (CV) of the biodiversity data was 2, 4, 8, or 16 times the CV of the cost data. To do so, we added a fixed quantity to the cost of each planning unit, which increased the mean cost without altering the standard deviation, thereby decreasing the CV. This quanitity (Δ_*cost*_) was chosen based on the following formula:

$${\Delta }_{cost}=\frac{C{V}_{relative}\cdot S{D}_{cost}}{C{V}_{benefit}}-{\mu }_{cost}$$

where *SD* is the standard devition, *μ* is the mean, *CV* = *SD*/*μ* is the coefficient of variation, *CV*_*relative*_ = *CV*_*benefit*_/*CV*_*cost*_, and Δ_*cost*_ is the amount added to the cost layer to achieve the desired relative CV of 2, 4, 8, or 16. Throughout this process, the benefit CV was held constant and measured as the average CV of the species occupancy layers. We then performed all of the prioritizations described above for each of the relative CV values. In each case (with and without cost data; with and without biodiversity data), we produced cost-benefit curves illustrating the cost, as a percentage of the total cost of the entire study region, to achieve a given occupancy target. More efficient solutions are depicted with steeper cost-benefit curves and reach a higher occupancy target for lower cost. As such, we used the area under the cost-benefit curves as a metric of the efficiency of prioritization approaches across all occupancy targets.

## Results

Land cost and biodiversity feature data varied widely across our study area for both focal species group. Planning unit costs varied over 8 orders of magnitude, from $744 to 44.1 billion dollars per km^2^ (mean = $78 ± 565 million; CV = 7.25). The coefficients of variation in species occupancy probability predictions ranged from 0.407 to 1.415 (Supplementary Material Table 1). On average, the predicted occurrence of human-associated species was positively related to land cost (r_cost_ = 0.083 ± 0.094; mean ± standard deviation), whereas forest species occurrence declined with land cost (r_cost_ = −0.066 ± 0.053; mean ± standard deviation; Supplementary Material Table 1).

**Figure 2:**
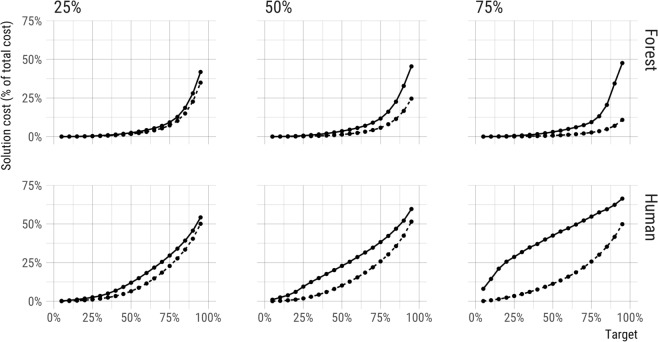
Using biodiversity feature data in conservation prioritization (dashed line) improved the efficiency of meeting conservation targets as compared to using parcel cost alone (solid line). Cost-only solutions were derived purchasing land from least to most expensive until targets were met (C-rank prioritization; biodiversity data only used to determine when targets were meet; see Methods). Only parcels meeting the indicated occupancy threshold (25%, 50%, or 75%) were used to ensure the selection of parcels where species were very likely to occur.

**Figure 3:**
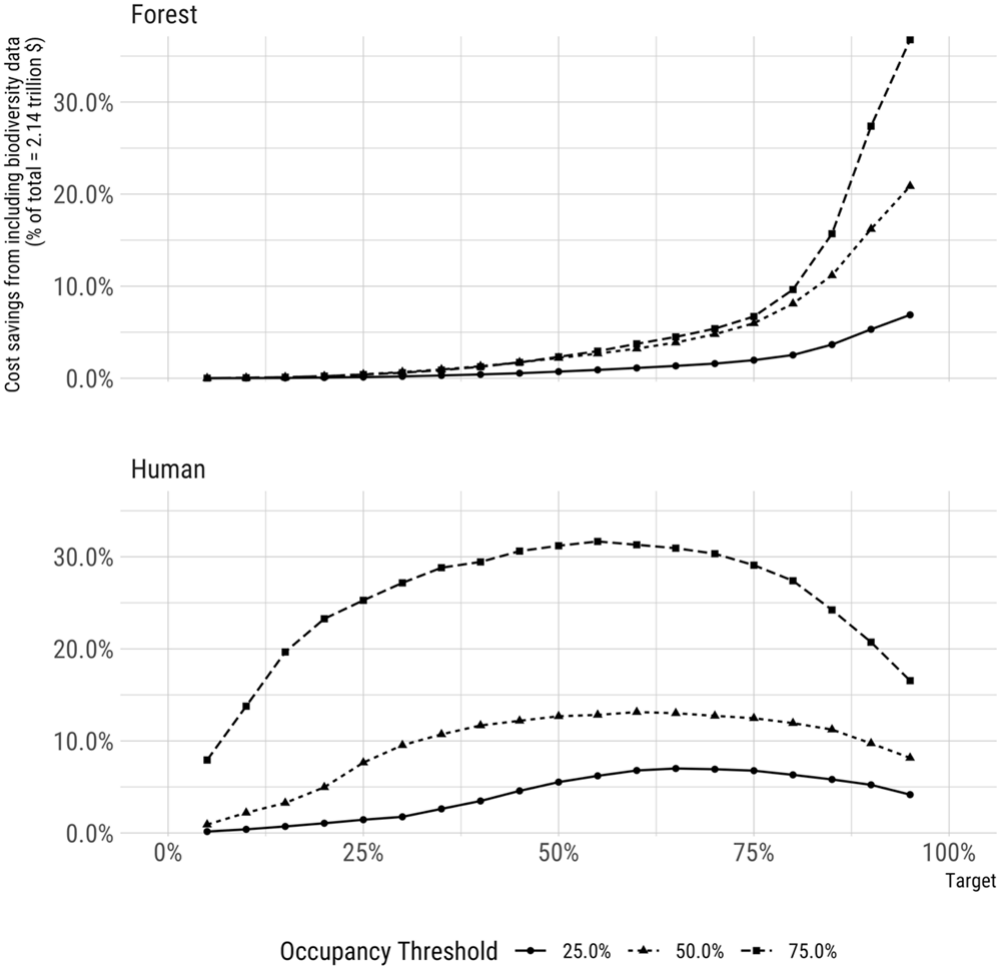
Cost savings varied widely across conservation targets and occupancy thresholds when including or excluding biodiversity feature data in Marxan-like prioritizations for forest and human-associated species. Restricting the prioritization to only select higher quality habitat (i.e. increasing the occupancy threshold), led to greater cost savings from including biodiversity data. Similarly, higher occupancy targets also led to an increase in the cost savings from including biodiversity data.

**Figure 4:**
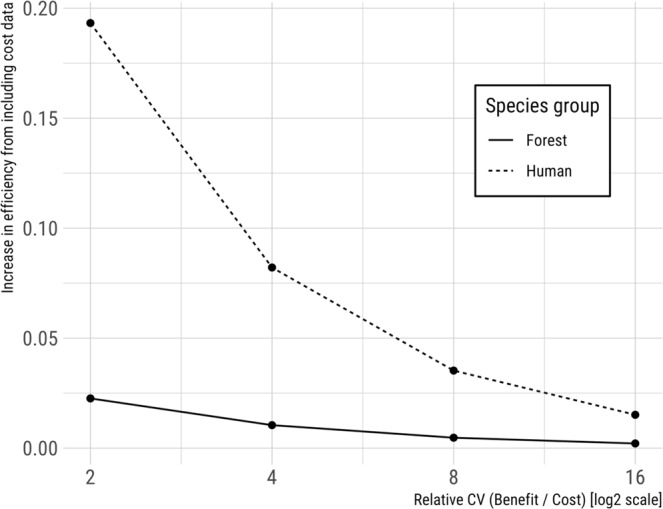
Fractional gain in efficiency when using both cost and biodiversity data, as compared to biodiversity data alone, declined as the relative variability of costs decreased. Human-associated species (dashed line) experienced a greater gain in efficiency from incorporating cost data than forest-associated species (solid line).

**Figure 5:**
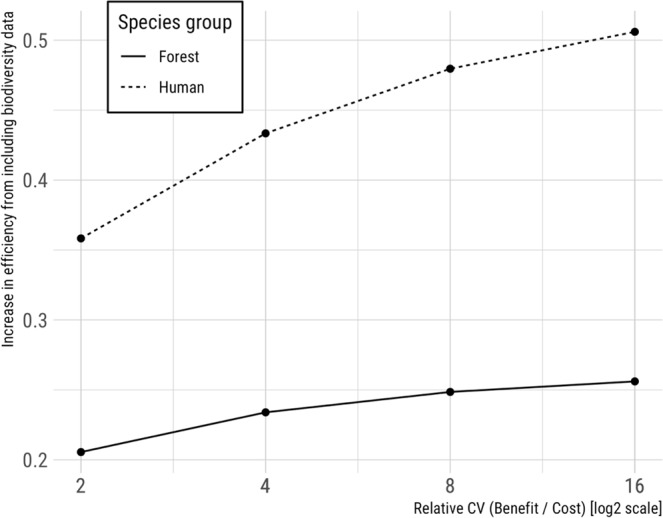
Fractional gain in efficiency from including biodiversity data in addition to cost data, compared to cost data alone, increased as the relative variability of biodiversity data increased. Human-associated species (dashed line) experienced a greater gain in efficiency from incorporating species data than forest-associated species (solid line).

Contrary to the assumption that biodiversity feature data reliably enhances the efficiency of spatially-optimized conservation plans, we found that the relative value of cost and biodiversity data varied by context. First, the value of biodiversity data and efficiency of solutions increased as planning efforts adopted more ambitious conservation targets, and/or became more restrictive by raising the threshold for occupancy, or habitat suitability (Figs 2, 3). Second, although incorporating land cost in prioritizations tended to make scenarios more cost-effective, efficiency gains declined as the relative variability of biodiversity feature to land cost data increased (Fig. 4, Supplementary Material Fig. 2). Third, we observed that biodiversity data tended to drive solutions more so when spatial variation in biodiversity feature data was high, relative to spatial variation in cost data (Fig. 5, Supplementary Material Fig. 3). These relationships support our expectation that the most variable data layer was likely to be most influential of optimized solutions.

The influence of biodiversity feature and land cost data on solutions also differed among focal species as a consequence of underlying correlations between species occurrence and land cost. For example, human-associated birds were much more likely to occupy land that varied greatly in cost than did species relying on mature forest. Although human-associated species are not often targeted for conservation, there are many instances where species of conservation concern are likely to occur in high-cost landscapes (e.g., Coastal California Gnatcatcher, *Polioptila californica californica*^31^). Prioritizations for such ‘cost-correlated’ species were most efficient when both land cost (Fig. 4) and biodiversity feature data (Fig. 5) were incorporated. In contrast, gains in efficiency achieved by including land cost and/or biodiversity feature data were more modest for mature forest species, whose predicted occurrence was not strongly correlated with variation in land cost in the landscape we examined.

## Discussion

Biodiversity feature and land cost data are frequently used to prioritize portfolios of sites potentially capable of achieving conservation goals at the lowest land and/or management costs. We estimated the relative influence of biodiversity and land cost data empirically and illustrated the effect of spatial variation in cost and biological data by contrasting spatially-optimized solutions to scenarios including a wide range of habitat suitability targets and thresholds. Despite some contextual effects, four rules-of-thumb emerged from our analyses of these effects.

First, we found that including land costs in spatial prioritizations led to more efficient solutions in almost all cases. Consideration of land or opportunity cost has been widely shown to improve cost-efficiency of biodiversity conservation and/or reduces negative impacts on extractive and recreational sectors^32,33^. The value of cost data was similarly demonstrated in a review of global conservation decisions for seven taxonomic groups, for which biodiversity data were typically less influential than socioeconomic concerns^22^. Yet despite the fact that a vast majority of conservation professionals favorably regarded the inclusion of cost-effectiveness in planning exercises, most consider cost to be less important than other program design elements^34^ and, hence, seldom include cost as part of return-on-investment evaluations^35^. Indeed, a recent survey of individuals conducting spatial prioritizations showed that only one-quarter to one-third of prioritizations incorporated land value or cost of implementation^14^, suggesting a potential disconnect between motivation and practice in optimization exercises. One barrier to including cost may be the highly variable and aggregated ways that costs are estimated and/or reported^36^.

Second, biodiversity feature data became more influential of scenario outcomes as conservation targets became more ambitious (e.g., scenarios protecting 75% vs. 25% of suitable habitat; Fig. 5). This finding is interesting because conservation targets vary widely in practice; for example, the Convention on Biological Diversity aims to protect 17% of terrestrial ecosystems, whereas the Nature Needs Half movement aims to conserve 50% of 846 ecoregions globally (e.g., natureneedshalf.org). Still higher targets may be applied to species of particular concern to conservation, such as endemic, range-restricted, or critically endangered species.

Third, the value of biodiversity feature data tended to increase with thresholds used to identify suitable habitat (e.g., probability of occupancy ≥75% vs. 25%; Fig. 3), underscoring the potential influence of precision in maps used to set thresholds for suitable habitat. For example, uniform range maps (e.g., International Union for the Conservation of Nature (IUCN), BirdLife International) are widely used in conservation prioritization, but may contribute little spatial variance when used as biodiversity feature data. In contrast, improvements to uniform, expert-elicited, and other course-scale map products are occurring rapidly as citizen-science data are used to enhance existing and create new map products based on multi-species assemblages (e.g.^4,12,13,37,38^).

Fourth, our most general finding was that the value of biodiversity feature or land cost data depended on its relative variability (*CV*_*relative*_) and relationship to each other, and on the extent to which species occurrence patterns were correlated with spatial variation in land cost. As variability in land cost increased relative to variability in biodiversity data, cost increasingly drove solutions and vice versa – a finding that is consistent with Ferraro^18^ and Naidoo and Adamowicz^19^. Land cost had particularly strong effects on prioritization scenarios targeting ‘cost-correlated species’, i.e., species whose probability of occurrence increased in areas with high mean and variance in land cost. These effects appeared as comparatively larger efficiency gains in human-associated (positively correlated to cost) than forest-associated birds (weakly negatively correlated to cost). Conversely, when biodiversity features and costs were negatively correlated in space – as was the case for forest birds in our study, cost had less influence relative to biodiversity data alone. Other empirical studies have also found cost data to be more variable than biodiversity feature data, often by several orders of magnitude^16–20^. Perhans *et al*.^22^ reported that ecological data tended to be more variable than cost data when selecting among parcels of similar type and value. Taken together, these results and our own suggest that spatial variation in feature data can be used to anticipate its influence on optimized solutions to complex planning problems and, potentially, to evaluate the marginal value of ‘better’ data given the additional costs or effort required to collect it.

Spatial prioritizations are increasingly used to guide conservation and a recent survey showed that 74% of prioritizations intended for implementation produced action on-the-ground^14^. Because prioritization exercises inform real-world decisions, understanding the manner in which solutions are influenced by the types of data layers included is imperative. We showed that incorporating cost data greatly improved the efficiency of conservation planning solutions, particularly when biodiversity feature and cost data were positively correlated in space (e.g., when target species occurrence increased with land cost), and when spatial variation in cost exceeded spatial variation in benefits. We further showed that biodiversity feature data exerted a greater influence on solutions as conservation targets and/or the minimum thresholds of habitat suitability were increased, especially in cost-correlated species. One challenge potentially arising for planners is that, in practice, spatial variation in land cost, though often easier to estimate than biodiversity features, frequently exceeds variation in biodiversity feature data, especially in areas dominated by humans^17,20^. Consequently, there may be cases where the marginal value of additional or more precise biodiversity feature data has little or no effect on optimized solutions. It is also the case that access to or the affordability of cost data varies regionally and can be very hard to estimate, such as when tenure is uncertain or contested. Nevertheless, we suggest that considering correlations between cost and benefit data and variability in them should help decision-makers prioritize investments in data acquisition and refinement when attempting to maximize efficiency in spatial prioritizations of land for conservation. Although we recognize that insights based upon case studies are not uniformly applicable to different regions or planning contexts, our study reveals several important lessons that should be considered as part of the planning process.

## Supplementary information

Supplementary Information
