# Supplementary material for: Tradeoffs in the value of biodiversity feature and cost data in conservation prioritization
Source: Sci Rep. 2019 Nov 4;9:15921. doi: 10.1038/s41598-019-52241-2 (PMC6828800; doi:10.1038/s41598-019-52241-2)
Supplement: Supplementary file 1 — Supplementary Information [file 41598_2019_52241_MOESM1_ESM.docx]

**Supplementary Information**

**Landscape covariates**

Because birds are well-known to respond to many fine and coarse scale habitat features (Lawler and Edwards 2006), we developed covariate descriptors of landscape condition and context using coarse (1km) and fine (100m) scale features to advance early work conducted at coarse scales alone (De Wan et al. 2009). For modelling species detection and occurrence, we chose candidate predictors based on their proven ability to predict species occurrence at site and landscape levels in similar exercises or regions (Guisan and Thuiller 2005, Jewell et al. 2007, Schuster & Arcese 2013). All covariate names appear in Table S2 and were derived from the following sources: (i) Terrain Resource Information Management (TRIM, <http://archive.ilmb.gov.bc.ca/crgb/pba/trim/specs/specs20.pdf>), (ii) Terrestrial Ecosystem Mapping (TEM) of the CDF Zone (MES 2008), (iii) National Hydrography Dataset (<http://nhd.usgs.gov/data.html>), (iv) National Land Cover Database 2011 (Homer et al. 2015), (v) Global Forest Change (Hansen et al. 2013), (vi) OpenStreetMap data extracts (<http://download.geofabrik.de/>, accessed 2015-09-17), and (vii) Species distribution maps for 21 tree species in the region (Wang et al. 2016, Schuster and Arcese, unpublished data). Our dataset comprised 37 predictor covariates of site and landscape condition, derived at each of 2160 avian point count locations. All covariates were created using Geospatial Modeling Environment (Beyer 2012) in conjunction with ArcGIS 10.1 (ESRI 2012) and R v. 2.15.2 (R Development Core Team 2012). Due to their widely varying scales, all covariates were standardized about their mean value, to ensure that importance was not driven by measurement scale (White and Burnham 1999).

**Occupancy and detection models**

All methods adhere to the best practices for using and applying eBird data (Johnston et al. 2019), and data and code used to generate occupancy maps can be found at a GitHub repository (https://github.com/ricschuster/Tradeoffs-biodiversity-cost).

We assumed no variation in site occupancy across sampling occasions to minimize model complexity, thus assumed a closed population for all species (Mackenzie et al. 2002). The R package unmarked v. 0.9-9 (Fiske and Chandler 2011) provided the framework for all species models, which necessarily include two parts: occupancy and detection (Mackenzie et al. 2002). To estimate detectability, we used seven observation specific covariates (observer ID, Julian date, time of day, count type, effort hours, distance travelled, number of observers). For each of 73 bird species we fitted detectability models (without parameterizing occupancy) using a machine learning algorithm developed to work with the unmarked package (Supplementary Material Appendix A; Schuster and Arcese, 2013) and then ranked each by AIC (Akaike 1974). We then ranked all candidate models by AIC and averaged those with $\Delta$AIC ≤ 7 from the top ranked one (Burnham and Anderson 2002). The covariates that were present in the averaged model were always included as parameters on the detectability side in future models. Next we used the same machine learning algorithm from above to model occupancy using the 37 predictor covariates introduced earlier. The algorithm combines the paradigms of stepwise regression and multi-model inference using all possible covariate combinations, in a way that lets set of covariates included grow based on AIC, while trying to avoid shortcoming of classical stepwise regression functions that might not lead to the optimal configuration of covariates included in a model. We created this algorithm in part due to computational limitations of a true multi-model inference approach, using all possible covariate combinations, as this would have required us to fit a total of 2^37 (~137 billion) models per species. To drastically reduce this number, we create the hybrid algorithm first developed in Schuster and Arcese, (2013). We again ranked all candidate models by AIC and averaged those with $\Delta$AIC ≤ 7 from the top ranked one (Burnham and Anderson 2002) to create our final averaged model for each of the 73 focal species.

**Expert elicitation and habitat associations**

We asked 11 professional ornithologists with >5 years of local experience to rank the likelihood of 47 species in 10 focal habitat types using photographic and text descriptions of herbaceous, shrub, woodland, wetland, four forest types (pole, young, mature and old), and 2 human-dominated habitats (rural, urban). Experts ranked species by association (low = -1, medium = 0 or highly associated = 1) with each of 10 focal habitat types in CDF habitats. We then averaged ranks to describe habitat associations for each bird and habitat type and created two community association scores indicative of Human-dominated and Old Forest habitat associations that were standardized between 0 and 1 by dividing through the maximum value possible, where:

$$OldForest=\frac{-2*Herb-1*Shrub-0.5*Pole+0.5*YFor+1*MFor+2*OFor}{7}$$

$$Human=\frac{2*URB+1*RUR}{3}$$

We selected two groups to illustrate contrasting scenarios – one in which species were positively associated with land cost (human-associated species) and one in which species were negatively or uncorrelated with land cost (forest species). For the Human-associated species, we selected the 10 species with the highest Human community association score: European Starling (*Sturnus vulgaris*), House Sparrow (*Passer domesticus*), Rock Dove (*Columba livia*), American Crow (*Corvus brachyrhynchos*), House Finch (*Haemorhous mexicanus*), European Collared Dove (*Streptopelia decaocto*), Anna’s Hummingbird (*Calypte anna*), American bushtit (*Psaltriparus minimus*), Barred Owl (*Strix varia*), and Stellar’s Jay (*Cyanocitta stelleri*). For the species associated with forest, we selected the 10 species with the highest Old Forest community association score: Pileated Woodpecker (*Hylatomus pileatus*), Brown Creeper (*Certhia americana*), Red-breasted Nuthatch (*Sitta canadensis*), Hairy Woodpecker (*Leuconotopicus villosus*), Red Crossbill (*Loxia curvirostra*), Pacific Slope Flycatcher (*Empidonax difficilis*), Pacific Wren (*Troglodytes pacificus*), Varied Thrush (*Ixoreus naevius*), Townsend’s Warbler (*Setophaga townsendi*), and Golden-crowned Kinglet (*Regulus satrapa*).

**References**

Akaike, H. 1974. A new look at the statistical model identification. IEEE Trans. Automatic Control **19**:716–723.

Beyer, H. L. 2012. Geospatial Modelling Environment (Version 0.7.2.1). (software). URL: http://www.spatialecology.com/gme.

Burnham, K. P. & Anderson, D. R. 2002. Model selection and multimodel inference: a practical information-theoretic approach. Springer Verlag, New York, NY.

De Wan, A, Sullivan, P. J. Lembo, A. J. Smith, C. R. Maerz, J. C. Lassoie, J. P. & Richmond, M. E. 2009. Using occupancy models of forest breeding birds to prioritize conservation planning. Biol. Conserv. **142**:982–991.

ESRI. 2012. ArcGIS 10.1 Economic and Social Research Institute Inc., Redlands, CA. http://www.esri.com/.

Fiske, I. J. & Chandler, R. B.. 2011. unmarked : An R Package for Fitting Hierarchical Models of Wildlife Occurrence and Abundance. J. Stat. Software **43**:128–129.

Guisan, A. &Thuiller, W. 2005. Predicting species distribution: offering more than simple habitat models. Ecol. Lett. **8**:993–1009.

Hansen, M. C. Potapov, P. V. Moore, R. Hancher, M. Turubanova, S. A. Tyukavina, A. Thau, D. Stehman, S. V. Goetz, S. J. Loveland, T. R. Kommareddy, A. Egorov, A. Chini, L. Justice, C.O. & Townshend, J.R.G. 2013. High-Resolution Global Maps of 21st-Century Forest Cover Change. Science **342**:850–853.

Homer, C. G. Dewitz, J. A. Yang, L. Jin, S. Danielson, P. Xian, G. Coulston, J. Herold, N. D. Wickham, & J. D. Megown, K. 2015. Completion of the 2011 National Land Cover Database for the conterminous United States—Representing a decade of land cover change information. Phot. Eng. Remote Sens. **81**:345–354.

Jewell, K. J. Arcese, P. & Gergel, S. 2007. Robust predictions of species distribution: Spatial habitat models for a brood parasite. Biol. Conserv. **140**:259–272.

Johnston, A., Hochachka, W.M., Strimas-Mackey, M.E., Gutierrez, V.R., Robinson, O.J., Miller, E.T., Auer, T., Kelling, S.T., Fink, D. 2019. Best practices for making reliable inferences from citizen science data: case study using eBird to estimate species distributions. bioRxiv, p.574392.

Lawler, J. J. & Edwards, T. C. 2006. A variance-decomposition approach to investigating multiscale habitat associations. Condor **108**:47–58.

Mackenzie, D. I. Nichols, J. D. Lachman, G. B. Droege, S. J. Royle, J. A. & Langtimm, C. A. 2002. Estimating site occupancy rates when detection probabilities are less than one. Ecology **83**:2248–2255.

MES. 2008. Terrestrial Ecosystem Mapping of the Coastal Douglas-Fir Biogeoclimatic Zone. Madrone Environmental Services LTD., Duncan, BC. Mandrone Environmental Services LTD., Duncan, BC.

Wang, T. Hamann, A. Spittlehouse, D. & Carroll, C. 2016. Locally downscaled and spatially customizable climate data for historical and future periods for North America. – PLoS One. https://doi.org/10.1371/journal.pone.0156720

White, G. C. & Burnham, K. P.1999. Program MARK: survival estimation from populations of marked animals. Bird Study **46**:120–139.

| **Forest species** | | | |
| --- | --- | --- | --- |
| **Common Name** | **Scientific Name** | **CV** | $\boldsymbol{r}_{\boldsymbol{cost}}$ |
| Hairy Woodpecker | *Picoides villosus* | 0.751 | -0.104 |
| Pileated Woodpecker | *Dryocopus pileatus* | 0.579 | -0.094 |
| Pacific-slope Flycatcher | *Empidonax difficilis* | 0.587 | -0.110 |
| Red-breasted Nuthatch | *Sitta canadensis* | 0.427 | -0.047 |
| Brown Creeper | *Certhia americana* | 0.522 | -0.081 |
| Pacific Wren | *Troglodytes pacificus* | 0.468 | -0.127 |
| Golden-crowned Kinglet | *Regulus satrapa* | 0.466 | -0.033 |
| Varied Thrush | *Ixoreus naevius* | 0.545 | -0.057 |
| Red Crossbill | *Loxia curvirostra* | 0.474 | 0.057 |
| Townsend's Warbler | *Setophaga townsendi* | 0.883 | -0.061 |
| **All forest (mean ± sd)** | | **0.570 ± 0.143** | **-0.066 ± 0.053** |
| **Human-associated species** | | | |
| Rock Pigeon | *Columba livia* | 1.047 | 0.110 |
| Eurasian Collared-Dove | *Streptopelia decaocto* | 0.990 | -0.034 |
| Anna's Hummingbird | *Calypte anna* | 0.811 | 0.161 |
| Barred Owl | *Strix varia* | 1.415 | -0.039 |
| Steller's Jay | *Cyanocitta stelleri* | 0.407 | -0.065 |
| American Crow | *Corvus brachyrhynchos* | 0.419 | 0.106 |
| Bushtit | *Psaltriparus minimus* | 0.922 | 0.176 |
| European Starling | *Sturnus vulgaris* | 0.702 | 0.086 |
| House Finch | *Haemorhous mexicanus* | 0.789 | 0.160 |
| House Sparrow | *Passer domesticus* | 0.993 | 0.167 |
| **All human (mean ± sd)** | | **0.850 ± 0.300** | **0.083 ± 0.094** |

**Supplementary Table 1.** Cell-wise coefficient of variation (CV) and correlation with land cost (Pearson’s correlation coefficient; $r_{cost}$) of each species’ occupancy probability layer.

| **Covariate** | **Buffer** | **Source** | |
| --- | --- | --- | --- |
|  |  | Canada | US |
| Barren | 100m, 1km | TEM: Cliff, Exposed Soil, Gravel Pit, Mine, Rock Outcrop, Talus , Mine Tailings | NLCD2011: Class 31 Barren Land |
| Cultivated | 100m, 1km | TEM: Cultivated Field, Cultivated Orchard, Cultivated Vineyard | NLCD2011: Class 81 Pasture/Hay, 82 Cultivated Crop |
| Forest 1 | 100m, 1km | TEM: Structural stages 4, 5 (Pole/Sapling, Young Forest) for Classes AB, AF, CS, DC, DF, DG, DO, DS, HD, HK, HM, HS, LC, RB, RC, RF, RK, RP, RS, RT, RV, SS | GNN Structure Maps: STRUCCOND Salpling/Pole, Small/medium tree |
| Forest 2 | 100m, 1km | TEM: Structural stages 6, 7 (Mature and Old Forest), Classes as Forest 1 | GNN Structure Maps: STRUCCOND Large tree, Large/giant tree from NLCD 2011: Forest |
| Herbaceous | 100m, 1km | TEM: Structural stages 1, 2 (Sparse and Herb), Classes as Forest 1 | NLCD2011: Herbaceous |
| Rural | 100m, 1km | TEM: Break water, Golf Course, Rural | NLCD2011: Class 21 Developed, Open Space |
| Savannah | 100m, 1km | TEM: Classes AM, BA, CB, DA, FC, GO, LM, OM, OR, QB, SC | GNN Structure Maps: VEGCLASS 3, 4; CANCOV_HDW < 70% from NLCD 2011: Forest |
| Shrub | 100m, 1km | TEM: Structural stage 3 (Shrub), Classes as Forest 1 | NLCD2011: Shrubland |
| Water | 100m, 1km | TEM: Lake, Reservoir | NLCD2011: Class 11 Open Water |
| Wetland | 100m, 1km | TEM: AS, BE, CD, CW, Ed01, Ed03, Em01, Em02, Em03, Em05, GB, LS, MU, OW, PD, RA, RI, Wb50, Wf51, Wf52, Wf53, Wm04, Wm05, Wm06, Wm50, Wm51, Ws50, Ws51, Ws52, YG | NLCD2011: Wetlands |
| Urban | 100m, 1km | TEM: Dam, Industrial, Railway Surface, Road Surface, Urban/Suburban | NLCD2011: Class 22, 23, 24 Developed, Low, Medium, High Intensity |
| Shannon Diversity Index | 100m, 1km | TEM | NLCD2011 |
| Perimeter to area ratio | 100m, 1km | TEM | NLCD2011 |
| Road high use | 100m, 1km | OpenStreetMaps types: bridge, bus_stop, construction, living_street, motorway, motorway_link, primary, primary_link, residential, secondary, secondary_link, service, tertiary, tertiary_link, trunk, trunk_link | |
| Road low use | 100m, 1km | OpenStreetMaps types: abandoned, bridleway, cycleway, footway, path, pedestrian, road, social_path, steps, track, trail, unclassified | |
| Crown Closure | 100m | Global Forest Change (Hansen et al. 2013) | |
| Nearest freshwater | - | TRIM | National Hydrography Dataset (http://nhd.usgs.gov/data.html) |
| Nearest road | - | OpenStreetMaps types excluded: abandoned, bridleway, footway, path, trail | |
| Nearest urban | - | TEM: Dam, Industrial, Railway Surface, Road Surface, Urban/Suburban | NLCD2011: Class 22, 23, 24 Developed, Low, Medium, High Intensity |

**Supplementary Table 2.** Description of data sources for covariates.

**
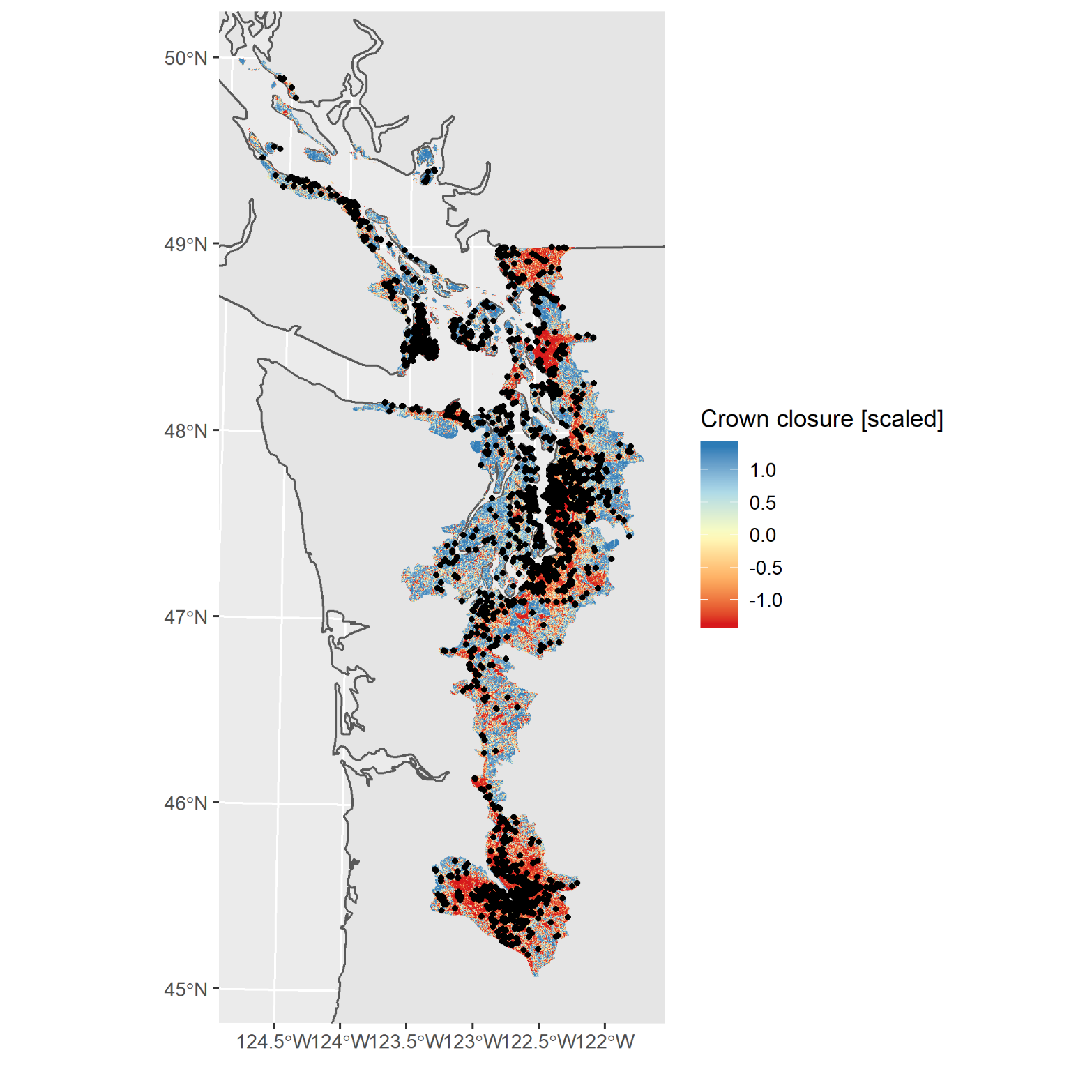
**

**Supplementary Figure 1. Point count locations and forest crown cover for survey data used to create occupancy models for biodiversity values.**

**
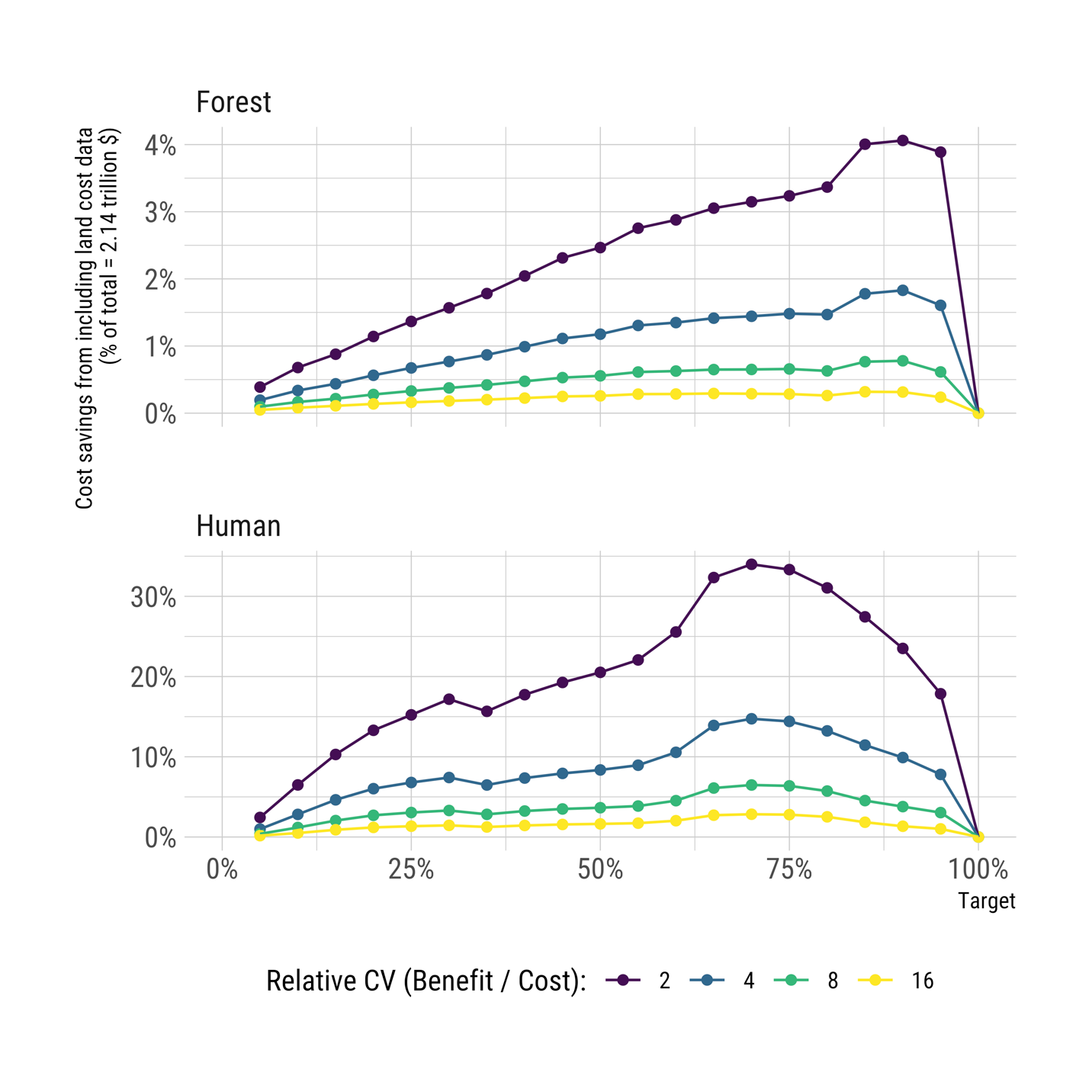
**

**Supplementary Figure 2.** The savings associated with using cost and biodiversity data, compared to using only biodiversity data, when prioritizing land across different representation target levels. These savings increased as the coefficient of variation of cost data increase relative to biodiversity data (decreasing relative CV). All prioritizations were performed using an occupancy threshold of 75%. Note that the relative CV is shown on a logarithm scale to highlight differences in color between the lines.

**
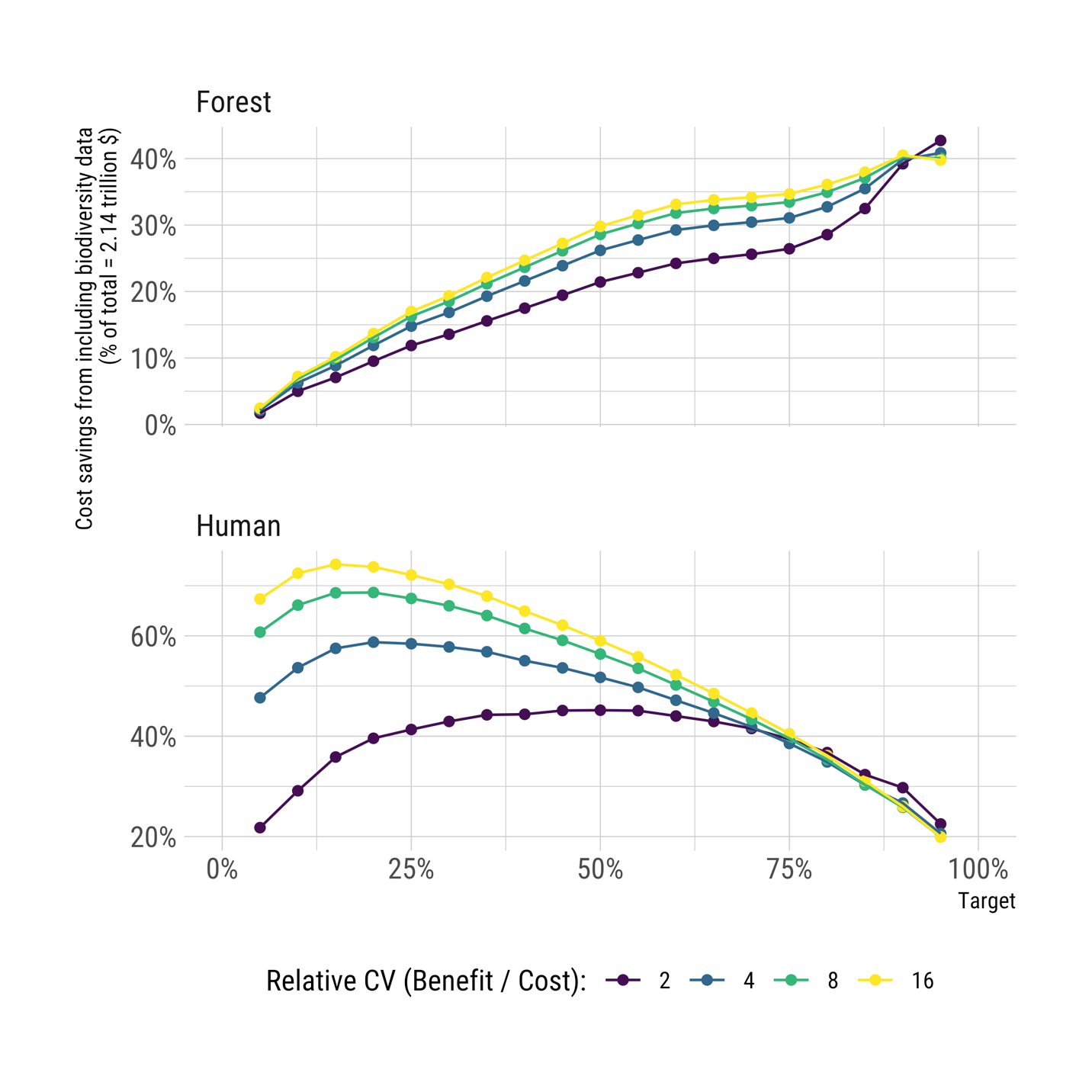
**

**Supplementary Figure 3.** The savings associated with using biodiversity and cost data, compared to using only cost data, when prioritizing land across different representation target levels. These savings increased as the coefficient of variation of savings data increase relative to cost data. All prioritizations were performed using an occupancy threshold of 75%. Note that the relative CV is shown on a logarithm scale to highlight differences in color between the lines.

Appendix A. R code of the function developed to fit occupancy models using a machine learning approach and to run the C-rank prioritization approach.

f.AICc.occu.sig <- function(start.model, blocks, max.iter = NULL, detocc = 1,

AICcut = 1, p.crit = 0.15, flag_se = FALSE, um.frame = "",

print.log = TRUE, ...) {

# f.AICc.occu.sig: a function for "stepwise" regression using occupancy models of package unmarked

# start.model: initial model e.g. occu(~1~1, UMF)

# detocc: if set to 1 (default) runs the function on the occupancy side; 2 does the detectability

# foreback: if TRUE covariates are allowed to be dropped; if FALSE only adding covariates allowed

# Parts based on Forward.lmer by Rense Nieuwenhuis (http://www.rensenieuwenhuis.nl/r-sessions-32/)

# with some additions by for Nick Isaac

# Author: Richard Schuster (mail@richard-schuster.com

# 08 October 2015

modlst <- c(start.model)

x <- 2

if (detocc == 1) {

coeff <- length(start.model@estimates@estimates$state@estimates)

} else {

coeff <- length(start.model@estimates@estimates$det@estimates)

}

best <- FALSE

model.basis <- start.model

keep <- list(start.model)

AICmin <- AIClst <- AICc(start.model)

# cutoff for when to exclude values

cutoff <- 20

# critical z value alhpa = 0.3 (1.036433); 0.15 (1.439531); 0.1 (1.644853); 0.05 (1.959964)

zc <- qnorm(1 - (p.crit / 2))

# Maximum number of iterations cannot exceed number of blocks, but this is also the default

if (is.null(max.iter) | max.iter > length(blocks)) max.iter <- length(blocks)

# Setting up the outer loop

for (ii in 1:max.iter) {

models <- list()

coeff <- coeff + 1

cnt <- 1

for (jj in 1:length(keep)) {

# Iteratively updating the model with addition of one block of variable(s)

for (kk in 1:length(blocks)) {

# check if blocks[kk] is already in the model, if so go to next kk

if (detocc == 1) {

if (blocks[kk] %in% names(keep[[jj]]@estimates@estimates$state@estimates)) next

} else {

if (blocks[kk] %in% names(keep[[jj]]@estimates@estimates$det@estimates)) next

}

if (detocc == 1) {

form <- as.formula(paste("~. ~. + ", blocks[kk]))

} else {

form <- as.formula(paste("~. + ", blocks[kk], "~. "))

}

if (class(dummy <- try(update(keep[[jj]], form, data = um.frame))) == "unmarkedFitOccu") {

flag <- 0

# check if model converged

if (dummy@opt$converge != 0) flag <- 1

# check if there is any NAN's in the SE's of the occupancy side

if (flag == 0 && detocc == 1) {

if (any(diag(vcov(dummy@estimates@estimates$state)) < 0) ||

any(sqrt(diag(vcov(dummy@estimates@estimates$state))) > cutoff)) {

flag <- 1

}

}

# check if there is any NAN's in the SE's of the detection side

if (flag == 0 && detocc == 2) {

if (any(diag(vcov(dummy@estimates@estimates$det)) < 0) ||

any(sqrt(diag(vcov(dummy@estimates@estimates$det))) > cutoff)) {

flag <- 1

}

}

# check for repeat models

if (flag == 0) {

for (bb in 1:length(AIClst)) {

if (round(AICc(dummy), digits = 6) == round(AIClst[bb], digits = 6)) {

flag <- 1

break

}

}

}

# Remove models where z < zc or SE = 0 for a beta > 0

if (flag == 0 && flag_se == TRUE) {

if (detocc == 1) {

if (any(abs(dummy@estimates@estimates$state@estimates[-1] /

sqrt(diag(vcov(dummy@estimates@estimates$state)))[-1]) <

zc)) {

flag <- 1

}

} else {

if (any(abs(dummy@estimates@estimates$det@estimates[-1] /

sqrt(diag(vcov(dummy@estimates@estimates$det)))[-1]) <

zc)) {

flag <- 1

}

}

}

}

else {

flag <- 1

}

# add dummy model to the model list if it passes all previous tests

if (flag == 0) {

models[[cnt]] <- dummy

modlst[[x]] <- models[[cnt]]

AIClst <- c(AIClst, AICc(models[[cnt]]))

x <- x + 1

cnt <- cnt + 1

}

}

}

if (length(LL <- unlist(lapply(models, function(x) {

AICc(x)

}))) == 0) {

break

}

keep <- list()

k <- 1

cont <- 0

# check for improvement in AIC, if none stop loop

for (mm in order(LL, decreasing = FALSE)) {

if (LL[mm] < AICmin + AICcut) {

if (detocc == 1) {

if (length(models[[mm]]@estimates@estimates$state@estimates) == coeff) {

keep[[k]] <- models[[mm]]

k <- k + 1

if (LL[mm] < AICmin) {

AICmin <- LL[mm]

cont <- 1

}

}

}

else {

if (length(models[[mm]]@estimates@estimates$det@estimates) == coeff) {

keep[[k]] <- models[[mm]]

k <- k + 1

if (LL[mm] < AICmin) {

AICmin <- LL[mm]

cont <- 1

}

}

}

}

else {

break

}

}

rm(models)

gc()

if (length(keep) == 0) break

}

## Create Model List

fitlst <- fitList(fits = modlst)

modsel <- modSel(fitlst, nullmod = NULL)

## Return the gathered output

return(list(model = model.basis, modlst = modlst, fitlst = fitlst, modsel = modsel))

}

##################################################

##################################################

##### Post-processing functions #####

##################################################

##################################################

occu.subset <- function(model, cutoff = 2) {

# subsetting the above function output so it can be used with

# model.avg from package MuMIn

# 2015-08-04

mdlst <- model

f.occ.aic <- unlist(lapply(mdlst, function(x) AIC(x)))

min.aic <- min(f.occ.aic)

f.occ.delta <- f.occ.aic - min.aic

return(mdlst[f.occ.delta < cutoff])

}

occu.rem.non.sig <- function(model, p.crit = 0.15) {

# critical z value alhpa = 0.3 (1.036433); 0.15 (1.439531); 0.1 (1.644853); 0.05 (1.959964)

zc <- qnorm(1 - (p.crit / 2))

model <- model$modlst

sig <- unlist(lapply(model, function(x) all(abs(x@estimates@estimates$state@estimates[-1] /

sqrt(diag(vcov(x@estimates@estimates$state)))[-1]) > zc)))

return(model[sig])

}

##################################################

##################################################

##### Functions from package MuMIn #####

##################################################

##################################################

.coefarr.avg <-

function(cfarr, weight, revised.var, full, alpha) {

weight <- weight / sum(weight)

nCoef <- dim(cfarr)[3L]

if (full) {

nas <- is.na(cfarr[, 1L, ]) & is.na(cfarr[, 2L, ])

cfarr[, 1L, ][nas] <- cfarr[, 2L, ][nas] <- 0

# cfarr[, 1L:2L, ][is.na(cfarr[, 1L:2L, ])] <- 0

if (!all(is.na(cfarr[, 3L, ]))) {

cfarr[, 3L, ][is.na(cfarr[, 3L, ])] <- Inf

}

}

avgcoef <- array(

dim = c(nCoef, 5L),

dimnames = list(dimnames(cfarr)[[3L]], c(

"Estimate",

"Std. Error", "Adjusted SE", "Lower CI", "Upper CI"

))

)

for (i in seq_len(nCoef))

avgcoef[i, ] <- par.avg(cfarr[, 1L, i], cfarr[, 2L, i], weight,

df = cfarr[, 3L, i], alpha = alpha, revised.var = revised.var

)

avgcoef[is.nan(avgcoef)] <- NA

return(avgcoef)

}

.makecoefmat <- function(cf) {

no.ase <- all(is.na(cf[, 3L]))

z <- abs(cf[, 1L] / cf[, if (no.ase) 2L else 3L])

pval <- 2 * pnorm(z, lower.tail = FALSE)

cbind(cf[, if (no.ase) 1L:2L else 1L:3L],

`z value` = z, `Pr(>|z|)` = zapsmall(pval)

)

}

add.coefmat <- function(object) {

is.arm <- ncol(object$msTable) == 6L && (colnames(object$msTable)[6L] == "ARM weight")

weight <- object$msTable[, if (is.arm) 6L else 5L]

object$coefmat <- .makecoefmat(.coefarr.avg(

object$coefArray, weight,

attr(object, "revised.var"), TRUE, 0.05

))

return(object)

}

R code for implementing C-rank prioritization.

#' Select sites via a C-Rank prioritization

#'

#' C-Rank prioritization is a greedy prioritization algorithm that selects site

#' starting at the cheapest and proceeding in order of increasing cost, until a

#' specified target level of representation is met for all of the input features.

#'

#' @param cost A RasterLayer object of the cost to protect each planning unit.

#' @param features A RasterStack object of biodiviersity features, with each

#' feature typically being the occupancy or abundance of a given species.

#' @param target A value between 0-1 specifying the percent of the total

#' biodiveristy value that must be met for all features.

#'

#' @return A logical vector specifying whether each planning unit has been

#' selected or not.

prioritize_crank <- function(cost, features, target) {

# convert to vectors and matrices, removing NA cells

cost_v <- cost[]

included_pu <- which(!is.na(cost_v))

cost_v <- cost_v[included_pu]

features_m <- t(features[][included_pu, ])

species <- names(features)

# order to choose PUs in

cost_order <- order(cost_v)

# decision variable

x <- rep(FALSE, length(cost_v))

# total amount

total_amount <- as.numeric(features_m %*% rep(TRUE, length(cost_v)))

# watched

spp_cost <- setNames(rep(NA_real_, length(species)), species)

spp_n <- setNames(rep(NA_integer_, length(species)), species)

for(i in seq_along(cost_order)) {

x[cost_order[i]] <- TRUE

# check representation

pct_rep <- as.numeric(features_m %*% x) / total_amount

target_met <- pct_rep > target

# keep track of cost associated with meeting each target

if (any(target_met & is.na(spp_cost))) {

cost_x <- sum(cost_v[x], na.rm = TRUE)

spp_cost[target_met] <- dplyr::coalesce(spp_cost[target_met], cost_x)

spp_n[target_met] <- dplyr::coalesce(spp_n[target_met], i)

}

# if all targets met, finish

if (all(!is.na(spp_cost))) {

break()

}

}

# prepare results

x_out <- rep(FALSE, raster::ncell(cost))

x_out[included_pu] <- x

x_out

}

R code for Marxan-like prioritizations using prioritizr

library(prioritizr)

# remove values from a raster below a given threshold

raster_trim <- function(x, cutoff, absolute = TRUE) {

# allow for layer specific cutoffs

if (length(cutoff) == 1) {

cutoff <- rep(cutoff, raster::nlayers(x))

}

# determine percentiles

for (i in seq.int(raster::nlayers(x))) {

if (absolute) {

thresh <- cutoff[i]

} else {

thresh <- raster::cellStats(x[[i]], function(r, na.rm) {

stats::quantile(r, cutoff[i], na.rm = na.rm)})

}

x[[i]][x[[i]][] < thresh] <- 0

}

return(x)

}

# cost: RasterLayer of planning unit cost

# features: RasterStack of occupancy for each species

# target: % representation target

# threshold: occupancy threshold, set occupancy to 0 below this level

solve_problem <- function(cost, features, target, threshold = 0) {

if (threshold > 0) {

features <- raster_trim(features, threshold)

}

problem(cost, features) %>%

add_min_set_objective() %>%

add_relative_targets(target) %>%

add_binary_decisions() %>%

add_gurobi_solver(gap = 0.01) %>%

solve()

}
